# Oil Motion Control by an Extra Pinning Structure in Electro-Fluidic Display

**DOI:** 10.3390/s18041114

**Published:** 2018-04-06

**Authors:** Yingying Dou, Biao Tang, Jan Groenewold, Fahong Li, Qiao Yue, Rui Zhou, Hui Li, Lingling Shui, Alex Henzen, Guofu Zhou

**Affiliations:** 1 Guangdong Provincial Key Laboratory of Optical Information Materials and Technology & Institute of Electronic Paper Displays, South China Academy of Advanced Optoelectronics, South China Normal University, Guangzhou 510006, China; douyingying@scnu.edu.cn (Y.D.); yueqiao@m.scnu.edu.cn (Q.Y.); zhourui@m.scnu.edu.cn (R.Z.); huili@m.scnu.edu.cn (H.L.); shuill@m.scnu.edu.cn (L.S.); 2 National Center for International Research on Green Optoelectronics, South China Normal University, Guangzhou 510006, China; jg@denk-werk.nl (J.G.); frank.Li@scnu.edu.cn (F.L.); alex-henzen@scnu.edu.cn (A.H.); 3 Van’t Hoff Laboratory for Physical and Colloid Chemistry, Debye Research Institute, Utrecht University, Padualaan 8, 3584 CH Utrecht, The Netherlands; 4 Shenzhen Guohua Optoelectronics Tech. Co. Ltd., Shenzhen 518110, China; 5 Academy of Shenzhen Guohua Optoelectronics, Shenzhen 518110, China

**Keywords:** oil motion, extra pinning structure (EPS), response time, electro-fluidic displays

## Abstract

Oil motion control is the key for the optical performance of electro-fluidic displays (EFD). In this paper, we introduced an extra pinning structure (EPS) into the EFD pixel to control the oil motion inside for the first time. The pinning structure canbe fabricated together with the pixel wall by a one-step lithography process. The effect of the relative location of the EPS in pixels on the oil motion was studied by a series of optoelectronic measurements. EPS showed good control of oil rupture position. The properly located EPS effectively guided the oil contraction direction, significantly accelerated switching on process, and suppressed oil overflow, without declining in aperture ratio. An asymmetrically designed EPS off the diagonal is recommended. This study provides a novel and facile way for oil motion control within an EFD pixel in both direction and timescale.

## 1. Introduction

**Figure 1:**
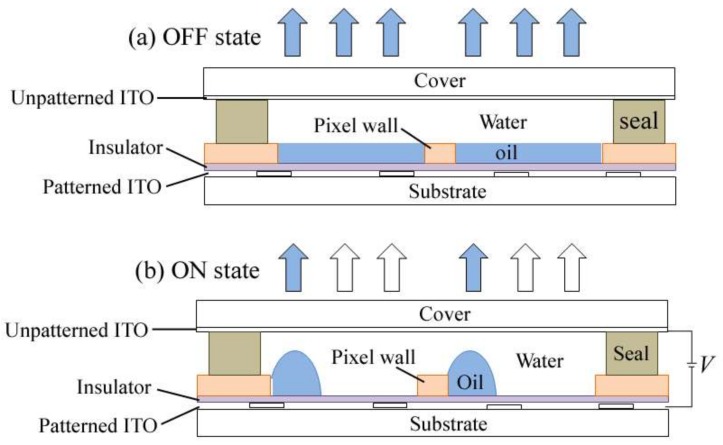
Working illustration of an electro-fluidic device (EFD). (**a**) OFF state without voltage; (**b**) ON state with a certain voltage.

Electrowetting has shown its power in manipulating micro-fluidics, which inspired applications like microfluidic chips [1,2] as well as electro-fluidic displays (EFD) [3]. To EFD, an oil/water two-phase microfluidic system, the oil motion control is the key for its optical performance, like gray scale, switching response, maximum aperture ratio, oil contraction direction, and so on [4,5].Therefore, the dynamic of oil/water interfacial movement attracts great attention in this field [6,7,8,9,10,11]. For a typical EFD pixel (Figure 1), which is sub-millimeterin size, the dominant driving forces for oil motion are recognized as interfacial tension and electrostatic force [12]. The interfacial boundary is normally described by the Young-Lippmann equation.

(1) $$\cos\theta ={\cos\theta }_{Y}+cU^{2}/2\sigma$$

where θ is the voltage-dependent contact angle in electro-fluidic display, θ_Y_ is the Young’s angle without voltage, *σ* is the interfacial tension between oil phase and water phase, *c* is the capacitance per unit area between the water phase and the electrode, and U is the applied voltage. A dimensionless number [7] is often used to describe the electric field induced driving force in an electro-fluidic device: $EW=cU^{2}/2\sigma$.

Without voltage (Figure 1a), oil covers the hydrophobic insulator surface inside the pixel, which is the off state of the EFD. When a voltage is applied to the electro-fluidic pixels (Figure 1b), the electrostatic force drives conductive liquid (water in our case), wetting the insulator surface, and pushing the oil motion. The oil motion during the on-switching process presents the optical response of the EFD, which could be characterized by the change in the white area percentage (WA) [13]. 

The most common used method for controlling oil motion in EFD is by introducing a “notch” in the pixelated electrode layer (a designed area without electrode). The artificially designed difference in the electric field distribution shows reasonable guidance in the final location of oil contraction for pixel switching-on [4]. However, the in-smooth electrode edge brought by “notch” may generate localized high Maxwell stress, which increasesthe risk of dielectric breakdown and oil motion disturbance [7,11,14,15]. On the other hand, the driving schemes with different rising gradients also show significant influence on oil motion patterns inside pixels. The contradiction between fast response and high aperture ratio is presented in [5]. Moreover, the reproducibility of this method of oil manipulation is not well proved. By our previous comparative studies of modeling and experiments [16,17], the initiation and development of oil motion during pixel switching-on are well understood. Three stages of oil motion during the switching-on process and two stages during switching-off process were experimentally proved and theoretically predicted. 

In this paper, we introduce an extra pinning structure (EPS) in an EFD pixel to control the oil motion. Here, we report the influence of the EPS distribution on the initiation and direction of oil motion, and consequently, the optical response for on and off switching of the EFD pixel. The mechanism behind the oil guidance by EPS was well discussed and confirmed by a series of optoelectronic measurements. This paper provides a novel and facile way for oil motion control, which opens a new perspective for the optimization of EFD optical performance in both uniformity and response. 

## 2. Materials and Methods

### 2.1. Chemicals and Materials

Patterned indium tin oxide (ITO)-coated glass with a 0.7 mm thickness and 100 Ω/□ resistance (Leaguer Optronics Co., Ltd., Shenzhen, China) was used as the addressed substssrate, and un-patterned ITO-coated glass (Guangdong Jimmy Glass Technology Ltd., Foshan, China) was used as the cover plate. The insulator employed in our experiments was AF 1600X (Chemours Chemical Co., Ltd., Shanghai, China). Pixel wall and the extra pinning structure (EPS) were made of a negative photo-resist from a local supplier by using an aqueous solution (KOH) as the developing solution. Twelve MΩ.cm DI water was used as the polar liquid for filling. Oil with 0.21 M purple dye dissolved in decane (C_10_H_22_) was used as the non-polar liquid. The electro-fluidic display was sealed with a pressure sensitive adhesive (PSA). 

### 2.2. Preparation of Electro-Fluidic Display with or without Pinning Structure

**Figure 2:**
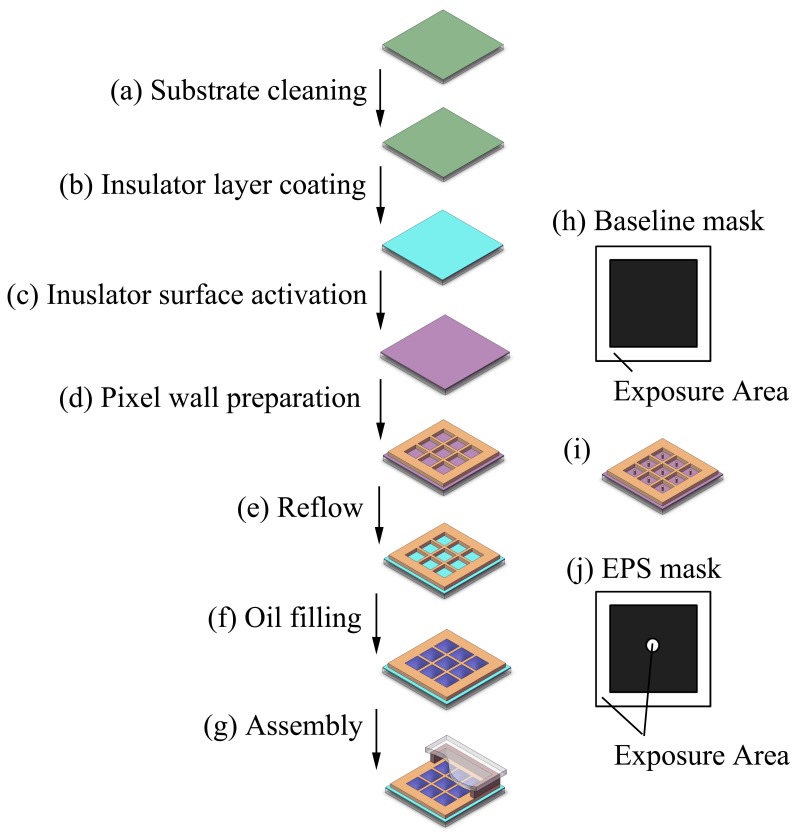
(**a**–**g**): Schematic diagram of the fabrication process of electro-fluidic displays; (**h**,**j**) are the top view images of the lithography masks of one pixel with (**j**) or without (**h**) extra pinning structure (EPS) design; (**i**): illustration of the pixels with central EPS design.

As shown in Figure 2a, the substrate glass with ITO layer was cleaned. Then amorphous fluoropolymer was spin-coated on the surface of the ITO layer of the glass substrate (Figure 2b) with a thickness of around 850 nm and baked on a hotplate for 3 min at 100 °C. A further curing process by heating in an oven at 180 °C for 30 min was conducted for completely removal of the solvent. Photoresist layer was spin-coated on the surface after a hydrophilic treatment of the fluoropolymer layer by a reactive ion etching process (Figure 2c). Then, a standard lithography process was carried on to fabricate the pixel wall and pinning structures (Figure 2d) with five different designed lithography masks with (central EPS as an example, Figure 2j) or without EPS design. The prepared pixels with central EPS design were illustrated in Figure 2i. After the lithography process, a thermal reflow process (Figure 2e) at 200 °C for 2 h was applied to restore the hydrophobic fluoropolymer layer [18]. The thickness of the pixel wall after the reflow process was around 5.6 μm, while the thickness of the pinning structure was 5.8 μm. The periodic length of each pixel was 165 μm, and the width of the pixel wall was 13 μm, while the diameter of the pinning structure was 15 μm. Then, along with the oil filling into pixel (Figure 2f), water and oil was sealed between the substrate and the cover by the pressure sensitive adhesive (Figure 2g). 

### 2.3. Oil Motion Observation

A high-speed camera (MIRO M110) with a speed of 2000 fps was used to observe the switching process of pixels at different voltages (25 V, 30 V, 35 V, and 40 V). The applied voltage was provided and controlled by a waveform generator (Agilent 33500B Series, Loveland, CO, USA) with a square wave of 5 Hz frequency.

## 3. Results and Discussions

### 3.1. Characterization of Pixel Structures Combined with An EPS

**Figure 3:**
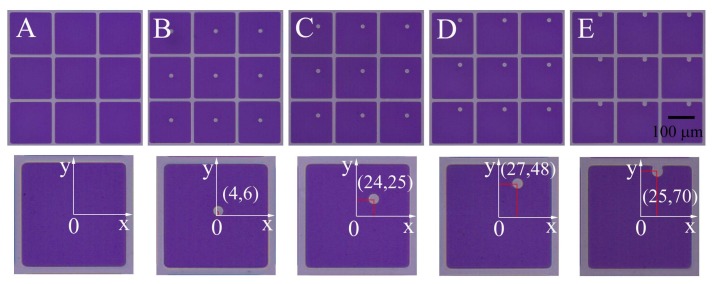
Pixel with different EPS distributions. The center of the pixel is chosen as the origin for the *x*, *y* axes. We use the value of (*x*,*y*) to describe the actual (measured) position of the EPS in μm. (**A**) the baseline case without EPS; (**B**–**E**) EPS with different locations.

Four types of EPS array were fabricated together with pixel wall by a one-step lithography process, as shown in Figure 3B–E. The (*x*,*y*) coordinates show well-controlled position and shape of the EPS distributed inside pixels. 

### 3.2. Optical Control by Pinning Structure

#### 3.2.1. EPS Guided Oil Motion during Pixel Switching-On

The placement of an EPS leads to a decrease of the pixel symmetry. According to our previous study [16], the pixel switching on process can be divided into three separate stages: oil film rupture, oil dewetting, and oil rearrangement, which could be described by wavelength selection [17], linear dewetting, and coarsening process, respectively. The oil rupture initiates the oil motion, while the pinning and capillarity effects guide the oil contraction process. The localized pinning effect introduced by EPS brings an asymmetric wavelength distribution possibility inside the pixel, which determines the rupture position of oil film (Table 1). Relative coordinates (*x*,*y*) referred to pixel center (0,0) are employed for a quantitative description of EPS position.Consistent with theoretical predictions, oil film rupture occurs at the EPS’ farther away side from pixel wall. The introducing of EPS shows well control of the oil rupture position in pixel.

**Figure 4:**
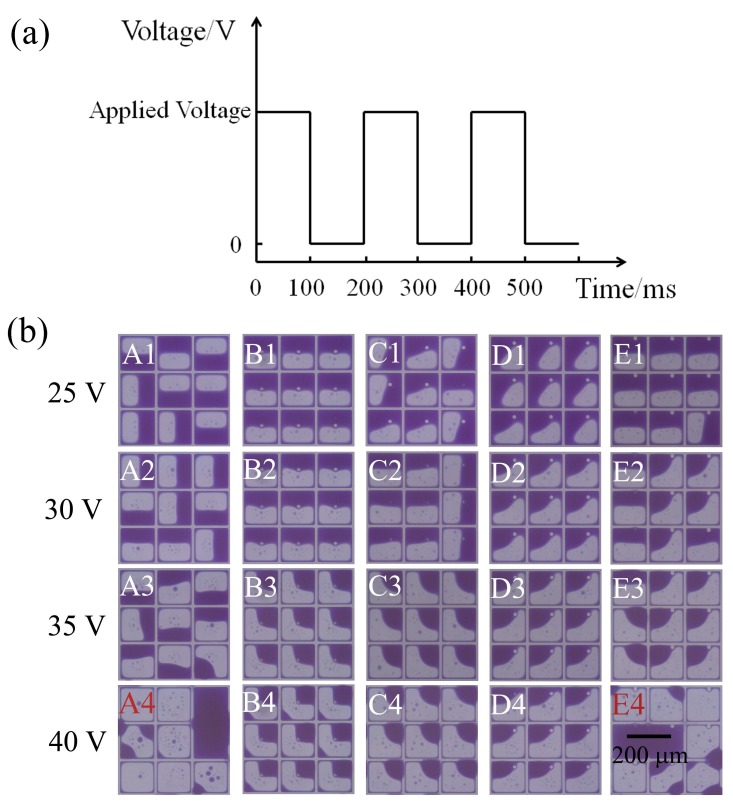
Oil motion control by EPS (**a**) DC driving waveform (5 Hz) with 100 ms pulse (**b**) pixel open states of different EPS distributions with increasing applied voltage.

After oil film rupture and oil dewetting, the oil rearrangement occurs, leading to the final oil open states. The movement of oil/water interface at the oil rearrangement process is driven by curvature related pressure differences between unequally sized oil drops. In contrast to the baseline case, high uniformity of oil states is observed in presence of an EPS as can be seen in Figure 4b (Column B–D). The asymmetry brought by EPS shows pretty good control of oil contraction direction, particularly at high aperture ratio state (high applied voltage), which can be explained by the localized extra pinning and capillary effects around the EPS. It does not show well-ordered oil contraction for sample E, caused by the location of the EPS already connected to pixel wall, which weakens the pinning and capillary effects. Samples A and E show obvious oil overflow at 40 V rather for samples B, C, and D, which indicates a properly located EPS can suppress oil overflow defect.

#### 3.2.2. EPS Influence on Pixel Aperture Ratio 

**Figure 5:**
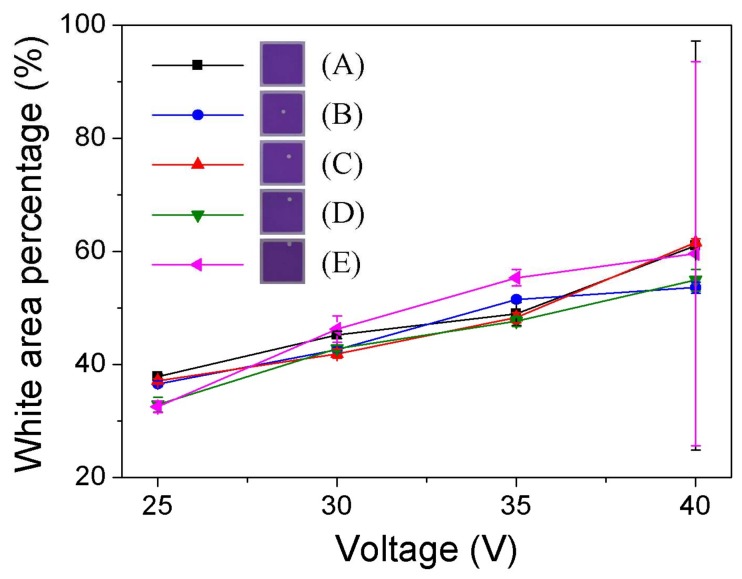
White area percentage (WA) at different applied voltages for pixels with different EPS designs. The aperture of nine pixels (3 × 3) was monitored for each sample. The error bar shows standard deviation (*σ*) in WA.

As shown in Figure 5, the white area percentage (WA) rises with increasing applied voltage for all the samples. The data indicates that the introduction of the EPS into the pixel brings no obvious influence in aperture ratio, which can be explained by the tiny relative volume and high transparency of EPS. With applied voltage increasing up to 40 V, a jump in error bar can be observed for samples A and E, which is caused by the occurrence of oil overflow corresponding to A4 and E4 in Figure 4b. Considering the function of the oil overflow suppression presented, pixels with EPS may gain a higher aperture ratio with high applied voltage. Because it was limited by the maximum output voltage (40 V), the enhancement of the aperture ratio by the EPS was not investigated here.

### 3.3. Response Time 

#### 3.3.1. Effect of EPS Location on Pixel Response

**Figure 6:**
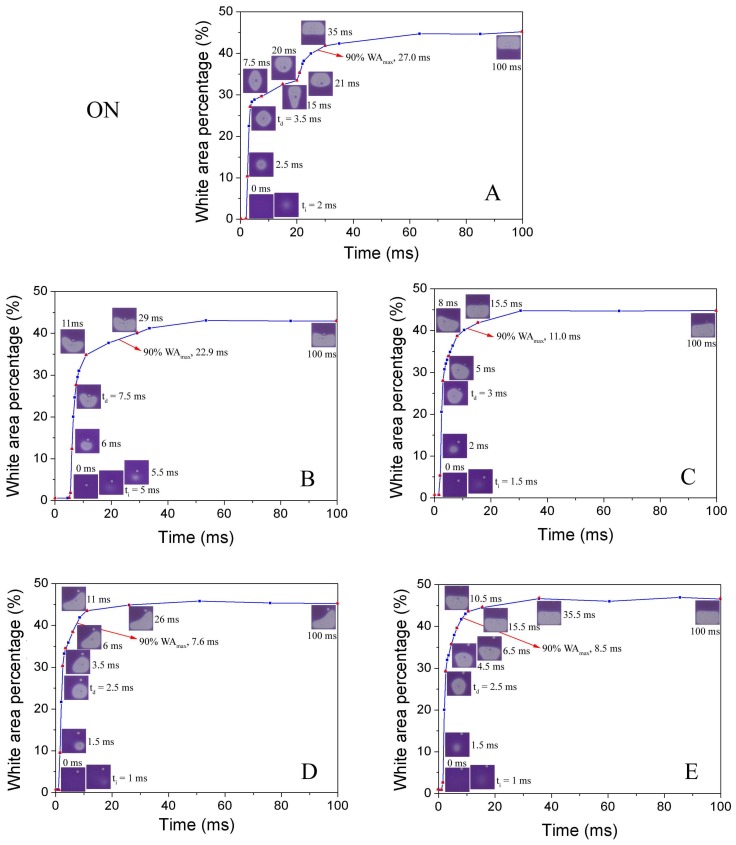
Comparison of optical response of pixel switching on process for base line pixel without EPS (**A**) and pixels with different EPS distributions (**B**,**C**,**D**,**E**), applied voltage = 30 V.

The response time is defined as the time from 0 ms point to the white area percentage reaching 90% WA_max_. The response time of the opening process is related to the three stages of electro-fluidic display (EFD): oil film rupture, oil dewetting, and oil rearrangement. Normally, the oil rearrangement stage is the most time-consuming process that dominates the response time of pixel switching on. The comparison of optical response of pixel switching on process for base line pixel and EPS located pixels is presented in Figure 6. The EPS located pixels show faster response, particularly for an off diagonal–designed EPS, compared to the pixels without an EPS (Figure 6 and Table 2). It is clear that only the asymmetrically located EPS (samples C, D, and E) brings slight shorter rupture time anda much fasteroil rearrangement process (Table 2), which emphasizing the artificial asymmetry in pinning and capillary is the mechanism behind the oil motion control by EPS. As a consequence, proper EPS distribution (diagonal, off-diagonal, and near-wall), such assample D (Figure 6D), provides the shortest on-switching response time (7.3 ms).

**Figure 7:**
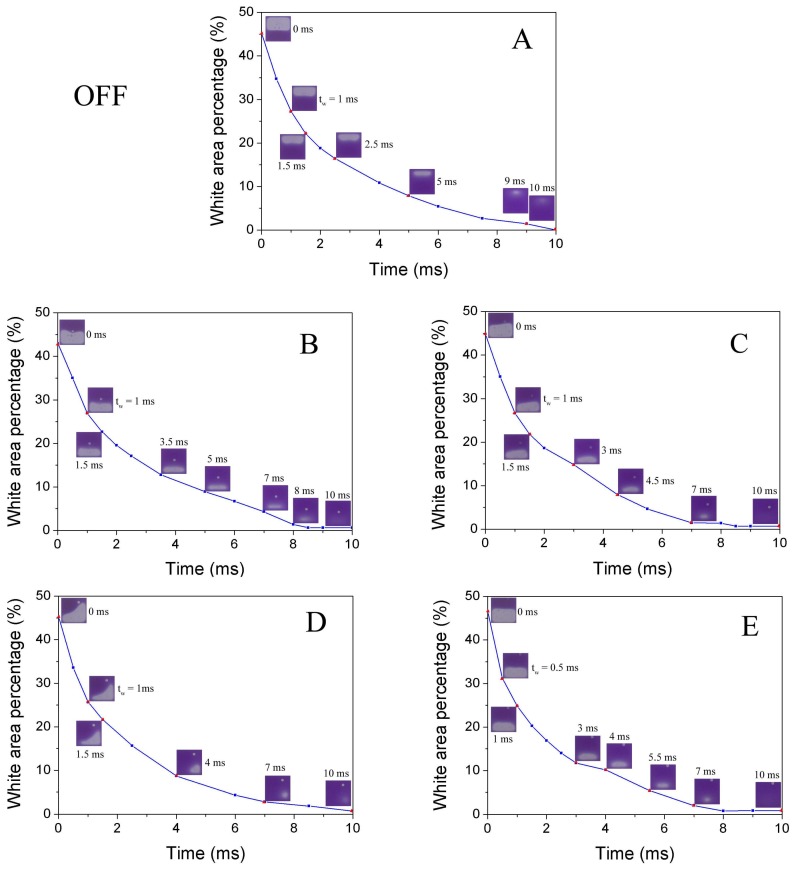
Comparison of optical response of pixel switching off process for base line pixel without EPS (**A**) and pixels with different EPS distributions (**B**,**C**,**D**,**E**), applied voltage = 0 V.

The switching-off process can be separated into two stages: oil wetting (t_w_) and oil reforming (t_off_). After switching-off, the contracted oil starts to spread spontaneously, which is driven by the stored surface potential converted from electric field energy [16]. When the merging of oil droplets occurs, an oil reforming process becomes dominating the off-process dynamics. The slow decay process can be described by a capillary surface reforming dynamics of thin films. The comparison of optical response of pixel switching off process for base line pixel and EPS located pixels is presented in Figure 7. It indicates that all the structures have a short wetting time (0.5–1 ms, Figure 7), and the oil reforming process is the critical factor of the response time for off switching. Considering the fact that the small volume of the EPS compare to pixel size, the extra capillary brought is relatively small. Thus, the introducing of an EPS should bring no obvious influence on off-switching response, which is proved by experimental data shown in Figure 7 and Table 2. 

#### 3.3.2. Applied Voltage Dependent Pixel Response

**Figure 8:**
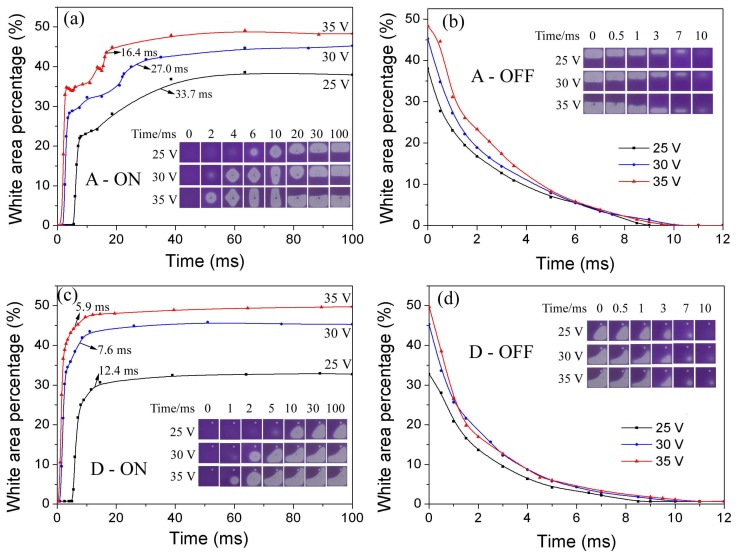
Voltage-dependent switching process of baseline pixels (**a**,**b**) and D-type pixels with off diagonal EPS (**c**,**d**). Inserted are the oil motion states in one pixel captured by high-speed camera.

The oil rupture vs applied voltage, the higher voltage applied on oil film, the stronger instability generated, and get a shorter rupture time [17]. The further dewetting process is dominated by voltage dependent electrowetting force, and the higher applied voltage gives higher moving velocity of three phase contact line [16]. The oil rearrangement time relates to the contact speed of the two drops, which is also highly dependent on applied voltage. Thus, we can see a shorter rearrangement time with a higher applied voltage. Generally, as presented in Figure 8, the samples with and without EPS all follow the applied voltage dependent rule proved in our previous work. A D-type pixel structure with an off-diagonal EPS obtains a simple oil rearrangement with oil rupture opposite to the corner where the oil is accumulated in the oil final open state, which explains the decrease in response time. Consistent with the previous analysis, EPS shows no big influence on off-switching response.

## 4. Conclusions

An extra pinning structure (EPS) was introduced into electro-fluidic displays (EFD) for the first time by a facile one-step lithography process. The distribution-dependent oil motion control function of an EPS located in an EFD pixel was fully discussed. The guidance of oil rupture and oil contraction by the EPS was investigated by a series of optoelectronic measurements. The EPS shows goodcontrol of the oil rupture position. The localized pinning and capillary effects are shown to effectively guide theoil contraction direction and significantly accelerate the switching-on process by simplifying the oil rearrangement stage. The off-diagonal EPS (D-type) gives the fastest on-switching response of 7.3 ms, without an obvious decline in the aperture ratio. Moreover, the EPS-introduced extra pinning effect provides obvious suppressing of oil overflow under high applied voltage. On the other hand, the introducing of EPS hasno obvious influence on the off-switching response. This paper provides a novel and facile way for oil motion control within an EFD pixel in both direction and timescale, which may inspire new applications in microfluidic manipulation. 

## 5. Patents

A Chinese patent was submitted in September 2017.

## Figures and Tables

**Table 1:** Oil rupture states with different EPS distributions. The unit of oil rupture position (*x*,*y*) is μm. R1 and R2 show the two oil rupture positions of E-type pixels.

No.	A	B	C	D	E
EPS distribution	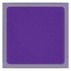	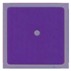	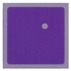	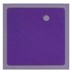	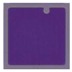
Oil rupture states	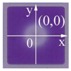	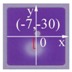	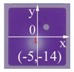	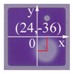	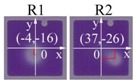

**Table 2:** The average data and the deviation of oil rupture time, response time, and off time for different EPS distributions with 12 pixels (4 × 3) for each sample.

No.	EPS Distributions	Oil Rupture Time (ms)	Response Time (ms)	Off Time (ms)
A	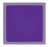	2.0 ± 0.0	23.5 ± 1.9	10.0 ± 0.1
B	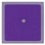	5.1 ± 0.3	21.7 ± 1.0	8.6 ± 0.2
C	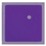	2.2 ± 0.3	10.9 ± 1.5	8.3 ± 0.3
D	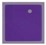	1.2 ± 0.3	7.3 ± 0.5	10.2 ± 0.6
E	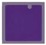	1.6 ± 0.2	8.6 ± 0.7	8.2 ± 0.6
